# Hypnotic analgesia reduces brain responses to pain seen in others

**DOI:** 10.1038/s41598-017-10310-4

**Published:** 2017-08-29

**Authors:** Claire Braboszcz, Edith Brandao-Farinelli, Patrik Vuilleumier

**Affiliations:** 1 Laboratory for Behavioural Neurology and Imaging of Cognition, Campus Biotech, University of Geneva, 1211 Geneva, Switzerland; 2 School of Psychology, University of Plymouth, Drake Circus, Plymouth, PL4 8AA United Kingdom; 3 Department of Anesthesiology, Pharmacology and Critical Care, University Hospital of Geneva, 1211 Geneva, Switzerland; 4 Swiss Center for Affective Sciences, University of Geneva, 1211 Geneva, Switzerland

## Abstract

Brain responses to pain experienced by oneself or seen in other people show consistent overlap in the pain processing network, particularly anterior insula, supporting the view that pain empathy partly relies on neural processes engaged by self-nociception. However, it remains unresolved whether changes in one’s own pain sensation may affect empathic responding to others’ pain. Here we show that inducing analgesia through hypnosis leads to decreased responses to both self and vicarious experience of pain. Activations in the right anterior insula and amygdala were markedly reduced when participants received painful thermal stimuli following hypnotic analgesia on their own hand, but also when they viewed pictures of others’ hand in pain. Functional connectivity analysis indicated that this hypnotic modulation of pain responses was associated with differential recruitment of right prefrontal regions implicated in selective attention and inhibitory control. Our results provide novel support to the view that self-nociception is involved during empathy for pain, and demonstrate the possibility to use hypnotic procedures to modulate higher-level emotional and social processes.

## Introduction

Studies of social cognition and empathy suggest that understanding another person’s emotional state recruits brain networks that mediate the same emotional state in the observer^1^. Thus, overlapping neuronal patterns are observed in the insula and cingulate cortex during the first-hand experience of pain and when viewing others in pain^2–5^ However, it remains unresolved whether neural mechanisms mediating self-nociception have a direct influence on empathic responses to others’ pain^6^. Patients with a rare congenital disease causing insensibility to pain were reported to show preserved activation of both insula and cingulate cortex to vicarious pain scenes^7^, suggesting that shared activation of these brain regions might not be necessary for pain empathy. Because these patients also recruited additional prefrontal regions^7^, it is possible that their intact empathy abilities relied on more cognitive processes subserving perspective taking and affective theory of mind^8^, allowing for indirect, top-down modulation of insula and cingulate areas. This would in turn be consistent with proposals that the latter regions encode more general saliency signals rather than pain-specific information^9^. Abnormal pain experience and developmental plasticity in patients with congenital analgesia might also account for a functional dissociation between vicarious and self-pain responses.

Here we investigated this issue by changing personal pain experience using a powerful and reversible modulation of nociception in healthy people. Self-perception of pain can be markedly reduced by induction of analgesia through hypnosis, a phenomena thought to imply top-down influences from selective attention and mental imagery^10^. Hypnotic analgesia is exploited in a variety of clinical settings, including surgery^11^. We therefore asked whether reduced sensitivity to pain under hypnosis would also affect brain responses to pain seen in others. Such an effect would support a direct functional link between self and vicarious experiences of pain.

We examined 20 healthy participants in two experimental protocols during a single fMRI session (3 were excluded due to movements and noisy datasets). The same tasks were conducted both in the normal state and after inducing hypnotic analgesia on the participants’ right hand (counter-balanced order). Hypnosis was administered by a trained anesthesiologist doctor. The first task was an event-related pain stimulation protocol in which individually thresholded thermal stimuli were delivered on the back of the right hand (noxious vs non-noxious in alternation, see Methods). Individual thresholds of pain were assessed both in the normal state and after induction of hypnotic analgesia. The second task presented participants with photographs of hands in a surgical context that depicted either painful or painless (but in both cases aversive) situations^3^. To ensure equal attention to images in all conditions, participants were asked to press a button using their left hand whenever a left hand was presented. Oddball pictures of various other body parts (without any emotional content) were also inserted in the task allowing us to test for non-specific effects of hypnosis on alertness and processing of salient stimuli. We predicted that viewing painful hand pictures should activate the brain pain matrix^2, 3^ but less so during hypnosis than normal state.

## Results

### Effect of hypnotic analgesia on brain responses during self-perception of pain

**Figure 1:**
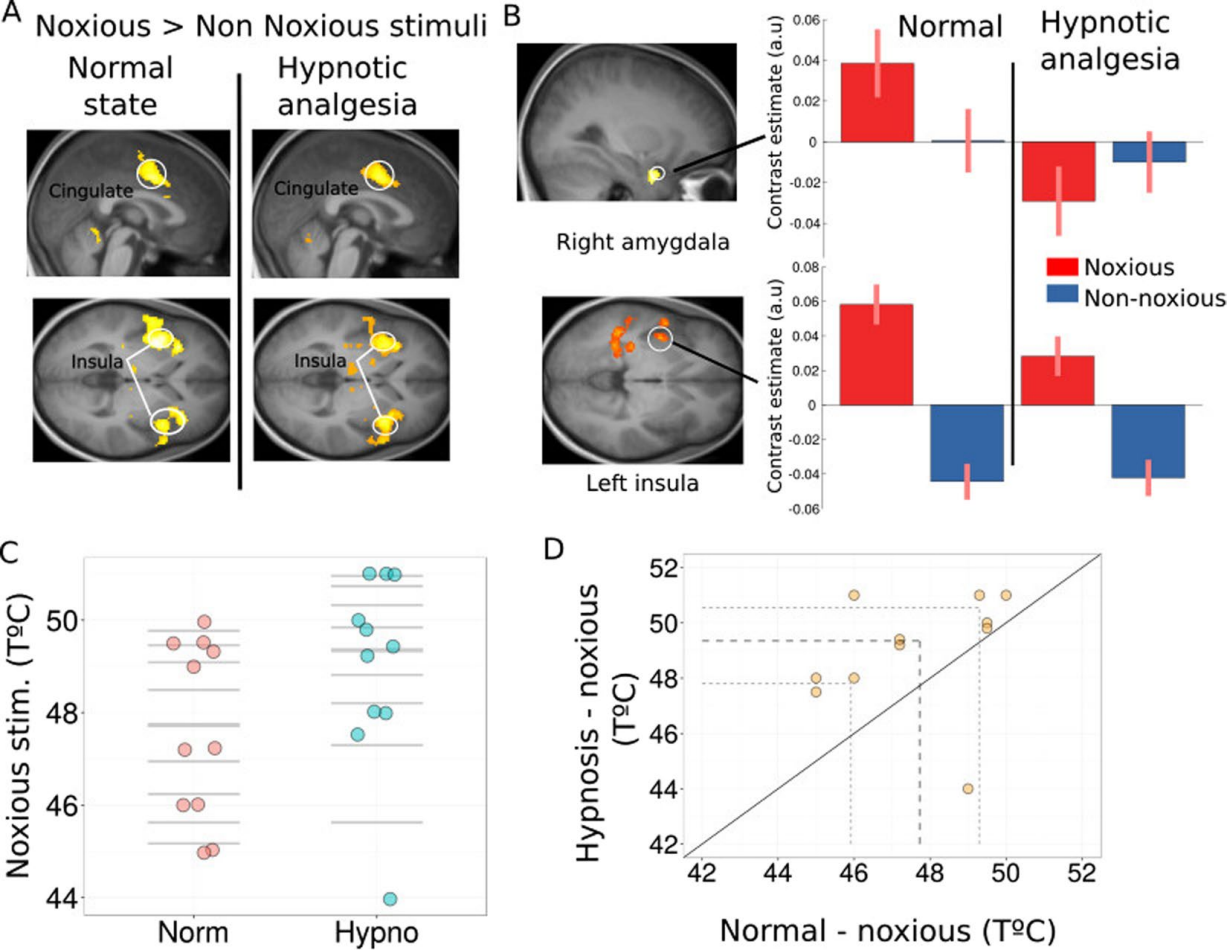
Brain response to felt pain. (**A**) Main effect of Noxious > Non Noxious thermal stimuli during normal and hypnotic analgesia conditions produced strong activation of the pain matrix (*p* < 0.05, fwe). (**B**) Activations to noxious stimuli were significantly enhanced during the normal compared to hypnotic conditions in the right amygdala (upper panel) and posterior and left insula (lower panel). Plots represent parameters estimates (beta values) extracted from these clusters for each type of stimuli in each condition. (**C**) Distribution of individually thresholded noxious temperatures in the normal and hypnotic analgesia conditions. The thick horizontal lines represent the median for each condition. (**D**) Scatterplot of paired observations for noxious stimuli temperature in hypnosis and normal conditions. The diagonal black line has a slope of 1 and intercept 0. The dashed grey lines mark the quartiles of the two conditions.

Painful thermal stimuli were adjusted to individual tolerance thresholds, using a staircase procedure similar to previous pain studies^5, 12, 13^. On each trial, participants reported whether heat was felt as painful or not, and their ratings were compared between the normal and hypnotic condition. Behavioral data from this task were not available for complete analysis in 6 participants due to technical recording problems. Fig. 1C and D show the distribution of temperatures for noxious stimuli during normal state and during hypnosis. The noxious stimuli temperature was higher during hypnosis than normal state (Fig. 1C). The individual threshold results for noxious temperatures revealed that during hypnotic analgesia the participants reported pain for temperatures that were on average 1.3 degrees higher than threshold temperatures defined during the normal state (wilcoxon signed rank test p = 0.03). This increase in pain tolerance was observed in all but one participants (see Fig. 1D) and cannot be attributed to habituation since the hypnotic and normal conditions were given in counterbalanced order across participants. Moreover, repeated short heat stimulation usually leads to pain sensitization rather than habituation^14^. This significant increase in temperature of the noxious thermal stimuli confirms the effectiveness of the hypnotic analgesia procedure.

At the brain level, as expected fMRI results showed significant activations in bilateral anterior insula (left Z score = 6.65; right Z score = 6.03) and cingulate cortex (Z score = 6.29) to noxious compared to non-noxious heat stimuli, as well as somatosensory cortex (Z score = 5.46), thalamus (Z score = 5.77), and PAG (Z score = 5.76) (Fig. 1A. and Supp. Table 1), during both the normal condition and hypnotic analgesia. However, contrasting painful heat stimulation in the normal versus analgesia condition revealed significant decreases in the left posterior insula under hypnosis together with the right amygdala (Fig. 1B). A formal interaction contrast (i.e. [nox > non-nox in Normal state] > [nox > non-nox in Hypnosis], see Table 1) confirmed significant changes in left insula (p = 0.029 fwe, SVC based on main effect of pain) but also in amygdala (p = 0.032 fwe, SVC). These results converge with the behavioral findings to show that hypnotic analgesia attenuated brain responses to self-experienced pain.

**Table 1:** Activation table for self-pain and seen pain.

Region at peak	x	y	z	size (k)	P	Z
**Self-pain**						
**Interaction [Noxious > Non-Noxious Norm]> [Noxious>Non-Noxious Hypno]**						
Post. Insula L.	−44	−8	−10	3	0.029**	3.36
Amygdala R.	22	−4	−18	43	0.032**	3.33
**Seen-pain**						
**Interaction [Painful images > Painless images. Norm]> [Painful images > Painless images Hypno]**						
Amygdala R.	32	−2	−24	30	0.016**	3.55
Ant. Insula L.	−26	22	8	134	0.04**	3.21
Ant.Insula R.	40	22	14	40	0.006	2.53
Thalamus R.	10	−6	2	185	<0.001**	4.67
PAG L.	−4	−20	0	98	0.001**	4.24
PAG R.	4	−18	−2	98	0.01**	3.7
SMA R.	14	−10	52	277	<0.001	4.24

*Fwe corrected whole brain; **Fwe corrected after SVC; PAG: periaqueductal grey; SMA: supplementary motor area; Ant. anterior; Post: posterior.

### Effect of hypnotic analgesia on brain responses to pain seen in others

**Figure 2:**
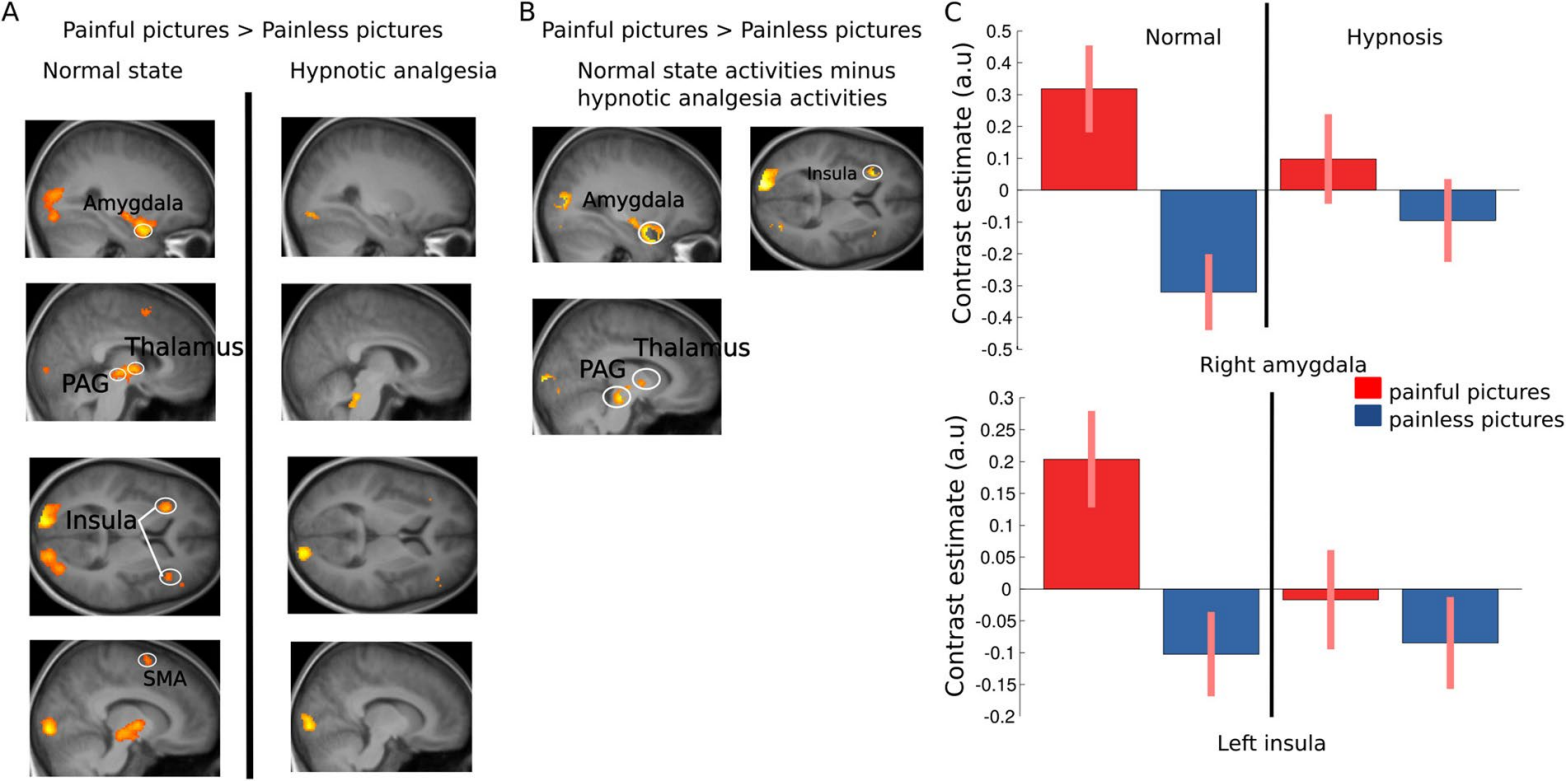
Brain responses to seen pain. (**A**) Brain areas responsive to the contrast Painful pictures > Painless pictures in the normal and in the hypnotic analgesia state. (**B**) Brain areas responsive to seeing pain activated specifically in the normal state and not in the hypnotic analgesia state. (**C**) Parameters estimate (beta values) for each type of stimuli and condition extracted from clusters in the right amygdala and left anterior insula.

In the empathy task, contrasting painful and painless hand pictures in the normal state (Fig. 2A) showed robust increases in the visual cortex (Z score = 7.25), amygdala (Z score = 5.30), thalamus (Z score = 5.96) and somatosensory cortex (Z score = 5.6 - all *p* < 0.01 fwe corrected for whole-brain, see Supp. Table 2), as well as anterior insula (Z score = 4.62), and periaqueductal grey area (PAG) (Z score = 4.31 - *p* < 0.05 SVC based on main effect of self-pain, see Supp. Table 2). Weaker activation was also found in supplementary motor area (SMA) (*p* < 0.001 uncorrected, p = 0.07 SVC, Z score = 3.92). This replicates previous work on empathy showing recruitment of pain processing networks when observing pain in others^3, 15, 16^.

During hypnotic analgesia, the same contrast yielded no significant activation in the pain matrix (see (Fig. 2A) and Supp. Table 2). To further investigate this difference, we tested for brain areas that were specifically activated in the normal state in response to scenes depicting hands in pain but not under hypnotic analgesia. To this aim we contrasted the effect of painful (versus painless) pictures in normal state after exclusive masking by the same contrast under hypnosis (using a liberal mask threshold of *p* < 0.05, Fig. 2B and see Supp. Table 2). This masking procedure allowed for a direct and stringent test of activations elicited in normal conditions only, with no such activations during hypnosis even at a trend level. Results confirmed differential responses of the right amygdala (Z score = 5.16), left anterior insula (Z score = 4.6), left and right thalamus (left thalamus Z score = 5.8, right thalamus Z score = 5.68), as well as the PAG (Z score = 4.48) when seeing hands in pain in the normal condition, but not present any longer under hypnotic analgesia. This effect was further confirmed by testing for the direct interaction contrast ([painful pictures > painless pictures in Normal state] > [painful pictures > painless pictures in Hypnosis]), which highlighted significant changes in the right amygdala (p = 0.016 fwe, SVC based on peak of main effect of painful pictures vs painless pictures, see Table 1) and left insula (p = 0.029 fwe, SVC based on peak of main effect), as well as in the PAG (p = 0.001 fwe, SVC based on peak of main effect) and posterior thalamus (*p* < 0.001 fwe, SVC based on peak of main effect). A weaker modulation was also observed in right insula (p = 0.006 uncorrected) and SMA (*p* < 0.001 uncorrected, see Table 1). The asymmetry of results between left and right insula might be due to our hypnotic induction that concerned the right hand only. Taken together, these findings indicate that analgesia produced by hypnosis did not only reduce felt pain to heat stimuli but also abolished empathy-related responses to seen pain in others (Fig. 2C and Table 1).

Interestingly, brain responses to the rare (oddball and ‘go’) stimuli did not show a similar reduction under hypnotic analgesia, confirming that the observed modulations were specific to pain recognition rather than due to a more general attenuation of insula and cingulate activity associated with the detection of salient stimuli^17^. In fact, during hypnosis, these rare stimuli produced significant increases in insula (Z score = 6.08) and cingulate cortex (Z score = 7.22), but also in SMA (Z score = 7.22) and sensorimotor areas (Z score = 6.32 - see Supp. Table 3). These differences were confirmed by a direct interaction contrast ([painful pictures in Normal state > painful pictures in Hypnosis] > [rare/oddball in Normal state > rare/oddball in Hypnosis]), showing activations in the left insula and in SMA (insula *p* < 0.05 Z score = 5.14, SMA *p* < 0.05, Z score = 4.18, both fwe corrected after SVC based on ROIs defined with the WFU PickAtlas). Weaker activations were also observed in the right amygdala(p = 0.001 uncorrected, Z score = 3.14). Thus, although rare stimuli by definition were relatively fewer than pain images, the lack of hypnotic effects on this condition cannot be attributed to fewer rare trials compared to the painful and painless images, since insufficient power would predict an opposite pattern with weaker activation under hypnosis.

Note that at the behavioral level, we did not find any significant difference in errors to the go-nogo task between normal state (mean = 3.8, sd = 2.4) and hypnotic analgesia (mean = 4.3, sd = 2.6, paired t-test: t = −1.02 p = 0.31). Likewise, no differences in reaction time (RT) were observed between the normal condition (mean RT: 1.80 sec, sd = 0.44 sec) and hypnotic analgesia condition (mean RT: 1.76 sec, sd = 0.47, t-test t = −0.7, p = 0.48). These results indicate that the hypnotic analgesia procedure did not affect general performance and arousal. Hence reduced brain responses to self-nociception and others’ suffering could not be explained by global slowing, apathy, or impaired attention.

### Anatomical substrates of felt and vicarious pain

To further verify the functional overlap between brain responses to self-experienced and vicarious pain, we also performed analyses on regions of interest (ROIs), predicted a priori based on previous studies of pain empathy. Using the thermal stimulation session, we defined a functional mask of clusters activated to painful heat in the normal state and then tested for responses to painful versus painless hand pictures with small volume correction for multiple comparisons. These analyses confirmed a significant overlap of direct and vicarious activations to pain stimuli in the left insula, PAG and right amygdala in normal state (*p* < 0.05 fwe, SVC). In contrast there was no significant effect of vicarious pain in these regions during hypnotic analgesia.

### Functional connectivity of areas responsive to vicarious pain

**Figure 3:**
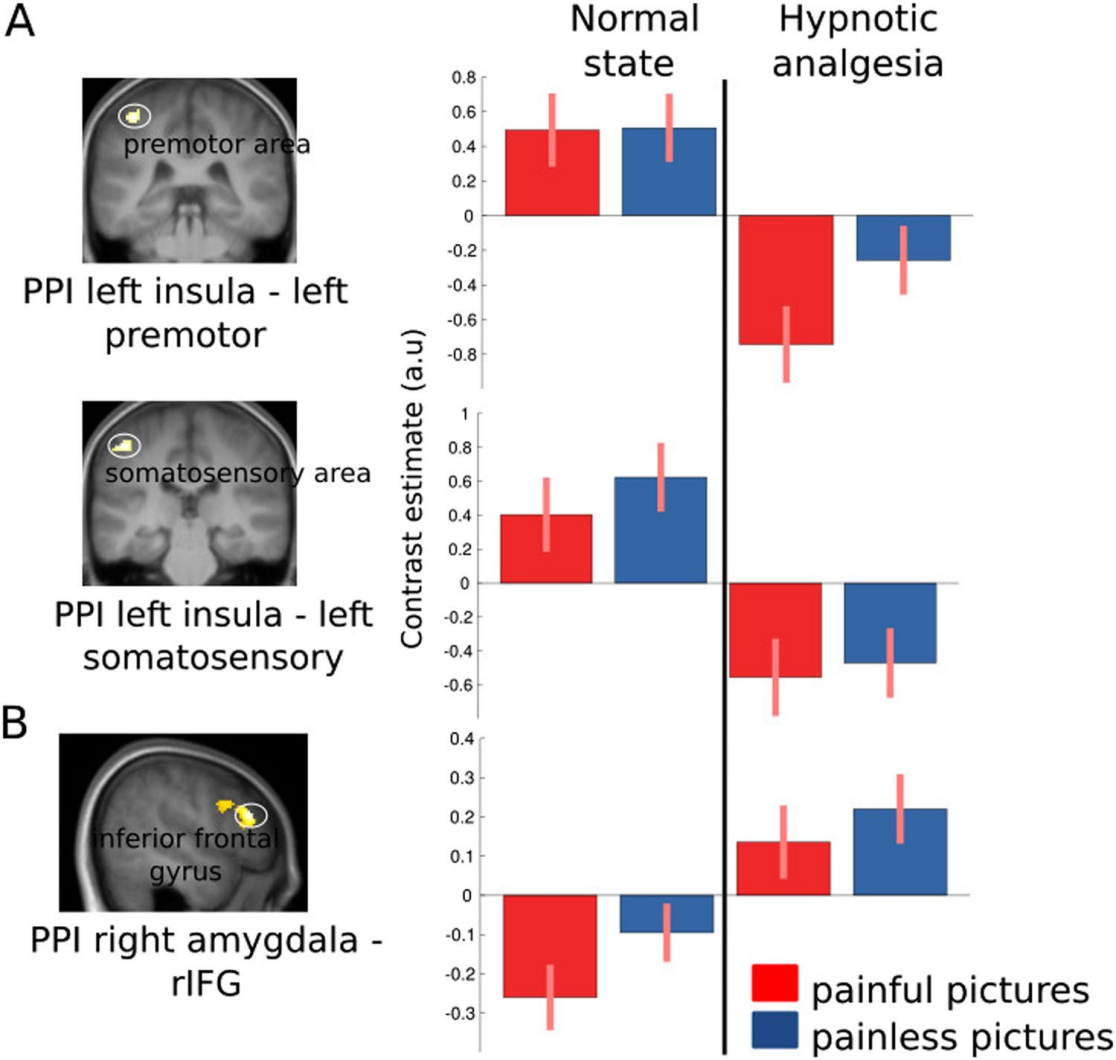
PPI analysis. (**A**) Less coupling between left anterior insula and left somatosensory and left premotor cortex during hypnotic analgesia. (**B**) Higher coupling between right amygdala and right inferior frontal gyrus.

Finally, we tested whether the above modulation induced by hypnosis were associated with changes in functional connectivity of pain-responsive areas. We conducted a psycho-physiological interaction (PPI) analysis of left anterior insula and amygdala activity during the empathy task, across both the normal condition and hypnotic analgesia. Results for the left anterior insula showed selective decreases in its connectivity with the left somatosensory and the left premotor cortex during hypnosis relative to the normal condition (*p* < 0.05, fwe, see Fig. 3A). Conversely, the right amygdala showed increased connectivity with the right inferior frontal gyrus (rIFG) during hypnosis (Fig. 3B).

## Discussion

We show for the first time that reduced pain sensation on one’s own hand as produced by hypnotic suggestion leads to attenuated activation of key emotional components within the pain matrix (including anterior insula, thalamus, periaqueductal grey (PAG) but also amygdala) when seeing other people’s hand in pain. There was no such decrease in other brain areas recruited during the task, or for other salient non-pain related stimuli. These changes were accompanied by reduced functional connectivity of the left anterior insula with sensory-motor areas, suggesting reduced impact of pain signals on covert movement preparation. Thus, hypnotic analgesia may not only reduce the emotional response associated with first-hand experience of pain but also modulates empathy reactions to the pain of others. Reduced activation in the amygdala in response to first-hand experience of painful stimulation under hypnotic analgesia confirms that hypnosis attenuated the aversive emotional components of self-experienced pain^18–21^. In turn, the right amygdala also exhibited attenuated responses to pain seen in others during hypnosis, together with increased functional connectivity with right prefrontal cortex. Activation in the PAG was also reduced during hypnotic analgesia in response to pain seen in others (though not to self-pain) suggesting again that hypnotic analgesia reduced negative emotional response^22^. This result may also suggest different mechanisms within the PAG in response to self and vicarious pain. Altogether, these findings clearly demonstrate that hypnotic analgesia can modulate activation in a wide brain network that is usually found to be recruited by both self and vicarious pain situations^2–4^.

Our results accord with the notion of a shared anatomical substrate between pain felt by oneself and empathy for pain seen in others^3, 4, 11^, but in addition reveal for the first time that this vicarious response is suppressed when self-pain experience is altered by hypnosis. These data therefore provide novel evidence that neural processes of empathy for pain are functionally equivalent, at least to some degree, to those engaged in the direct experience of pain, in agreement with embodied cognition account of empathy and emotional sharing^23^. Our findings converge with those from a recent study^24^ where placebo analgesia was found to reduce activations in the anterior insula and midcingulate cortex both in response to felt pain and empathy for pain. Taken together, these results confirm a recruitment of self-nociception mechanisms when appraising others’ pain, even though insula and cingulate activation may still arise in patients with a developmental insensitivity to pain, presumably through more cognitive top-down mechanisms of perspective taking^7^. Nevertheless, a recent fMRI study by ref. 25 argues for the existence of different patterns of activations for self and vicarious pain. Using a pattern classification approach, this study highlighted that brain areas involved when observing pain in others are more related to perspective taking than to somatic experience of pain. In ref. 25 study however, participants were explicitely instructed to imagine that the injury displayed in the picture was occurring to them or to judge wether a person was or not suffering, thus giving instructions that emphasized a perspective-taking approach to vicarious pain.

In our study, participants performed an indirect task (judging hand laterality) that allow for more “automatic” bottom-up empathic responses to pain cues in the pictures. Moreover, we also conducted a subsidiary analysis using pattern classification, which confirmed a consistent overlap of insula activations to felt and seen pain, similar to previous work with similar stimuli and task ref. 3, but unlike ref. 25. Differences in the instructions given to participants might therefore lead to differential processing of vicarious pain in different conditions, a variability that should be investigated in future studies.

Furthermore, we found less coupling between the anterior insula and somatosensory and premotor cortex when viewing pain in others in hypnotic analgesia state relative to normal state. Activations in somatosensory areas are not consistently reported in the litterature on empathy for pain and may relate to non-specific activations of the somatosensory networks upon viewing pictures of body-parts^4^. The decoupling between left anterior insula and left somatosensory areas might reflect altered emotional processing relative to the body (the right hand) during hypnotic analgesia^26^. The decreased coupling between the left anterior insula and the left premotor cortex that is also observed during hypnosis may relate to a similar mechanism.

On the other hand, increased coupling between amygdala and rIFG might reflect the well-known role of the latter region in executive control and inhibition for a wide range of behaviors, from motor responses^27^ to unwanted thoughts^28^ or memories^29^. Remarkably, recent studies on hypnosis found selective recruitment of the rIFG when motor paralysis is induced by hypnotic suggestion^10^ or when highly suggestible participants perform selective attention tasks^30^. It was suggested that the rIFG may serve general self-monitoring and attentional filtering functions that would allow internal mental representations, as those generated by hypnotic suggestion, to guide perception and behavior. Thus, higher coupling between the amygdala and rIFG under hypnotic analgesia in our study might reflect a top-down regulation of affective responses to seen pain, meditated by a modified representation of the self (i.e. numbness of the arm) induced by hypnosis. This result in itself provides novel insights into the role of right prefrontal cortex in hypnosis

Moreover, a recent neuroimaging study also suggested that hypnotic relaxation (without suggestion of analgesia) is associated with reduced activity in dorsal anterior cingulare cortex and increased functional connectivity between insula and dorso-lateral prefrontal cortex^31^. These changes might further contribute to reduced affective responses to both self and vicarious pain. Note however that in our paradigm, hypnotic analgesia did not modify responses to salient non-pain stimuli, indicating that hypnotic effects were not caused by a general reduction in saliency processing. Likewise, task performance and RTs showed no change between normal and hypnotic conditions, indicating that the reduced brain responses to both felt and seen pain could not be explained by lower vigilance or impaired attention.

One limitation of our study is that due to the specificity of our setting using hypnosis within the scanner environment, we could not obtain subjective ratings of pain intensity in relation to either the direct nociceptive stimulation or the observation of hand on a trial by trial bases. Not only this procedure may have weaken the hypnotic state induced in our participants, but also engender specific attentional biases and task demands confounds. Therefore, we cannot demonstrate a direct relationship between the subjective feeling of physical pain or emotional distress evoked by the seen or felt pain and the corresponding brain activations demonstrated with fMRI. Interestingly however, informal reports during post-experiment debriefings indicate that several participants found the go/nogo task performed in the empathy paradigm easier during hypnotic analgesia because they felt less emotionally disturbed by the view of hands in pain than during the normal state. Although anecdotal, these reports accord with the reduced activation in brain areas associated with aversive encoding of pain-related cues. In addition, previous research^2, 4, 16, 32, 33^ has consistently demonstrated a robust correlation between activation in the pain matrix areas and negative subjective feelings evoked by both seen and felt pain. Finally, although our painless control pictures contained aversive content (scalpels, surgery equipment), we did not directly compare neural changes associated with empathic responses under hypnosis with possible changes in non-pain emotional situations, which might potentially contribute to the observed effects through non-specific effects of hypnotic relaxation on anxiety. The selectivity of our findings for pain-responsive areas and our control conditions strongly suggest that such non-specific effects are not sufficient to explain our results, but future studies might usefully extend our approach to various other emotional conditions.

In conclusion, our results provide one of the most compelling evidence to date that pain empathy is not only anatomically, but also functionally related to the representation of pain experience within our own body. They also demonstrate for the first time that hypnosis can affect higher-level emotional and social processes in the brain – rather than more elementary perceptual or motor functions. This may have implications for the clinical use of hypnosis in domains associated with social and affective behaviors, and might even be usefully exploited in interventions aiming at regulating empathy in combination with other approaches^34^. Our novel results also highlight that such changes under hypnosis might be engendered through increased top-down signals from the rIFG, a region crucially implicated in self-monitoring and attention, which exhibited enhanced connectivity with right amygdala during hypnotic analgesia. In itself, this finding adds support to recent studies that point to a central role of the rIFG as the source of top-down mechanisms regulating behavior based on hypnotically-induced mental representations^10, 30^. As such, by unveiling neural circuits that determine how the brain flexibly encodes and interprets external inputs, our study provides novel insights illuminating both human social-affective abilities and the emerging neuroscience of hypnosis.

## Method

### Participants

Participants were recruited through advertisement in public areas of the University of Geneva and selected according to their score on the Harvard Hypnotic Suggestibility Scale^35^. Only participants scoring higher than 6 on a 12 points scale were invited to take part in the fMRI experiment. 20 participants were included in the study, all right-handed, with no history of physical or psychological disorder. All participants signed an informed consent form prior to participating in the study. The study was approved by the Geneva Cantonal Ethic Commission for Research on Human Beings (CER #14-040). All methods were performed in accordance with relevant guidelines and regulations. We excluded 3 participants from all analyses due to bad quality data (numerous movement artefacts). Analysis of the empathy task was thus done over 17 participants (11 females, mean age: 26 years old), among which 7 did the protocol with the hypnotic analgesia session first, followed by the normal session without hypnosis (vice versa for the remainder). Due to technical problems with the thermal stimulator, incomplete data were obtained for the pain localizer in 6 participants, who were then removed from this analysis. The order of conditions (hypnotic analgesia first or normal condition first) was counterbalanced across the remaining participants.

### Hypnotic analgesia procedure

Hypnosis was induced by a trained anesthesiologist doctor. Prior to participating to the experiment, all participants were screened using the Harvard Suggestibility Scale^35^ and where thus famialirized with classical hypnosis procedures. The hypnotic analgesia procedure used for this experiment followed the procedure used in clinical hypnosis to induce analgesia in patients. Hypnosis was induced while participants laid in the scanner, hearing the instructions through headphones. To assess the state of the participant, the hypnotist had access to online display of the participant’s breathing rate and could also see its right eye through an infra-red camera. The induction of hypnosis started with relaxation instructions in which the hypnotist guided the participant through pleasant mental images and sensations. The content of these mental images has been agreed upon with the hypnotist before the participant entered the scanner. For example the hypnotist could suggest the participant to imagine she/he was lying down on the warm sand of a quiet beach listening to the sound of the waves softly breaking on the shore. Once the hypnotist estimated a state of relaxation was reached, she started to introduce elements suggesting a feeling of sensation loss and numbness in the right hand and arm (i.e., “you start feeling as if you had been resting on your arm too long, as if your hand had been in the snow for a long period of time. Then your hand and arm become as the hand and arm of a statue, not moving, not feeling anything. And you can imagine you start wrapping this statue’s hand with layers of bandage, and even adding a glove over it. “). Hypnotic analgesia when participants were lying in the scanner, just prior to starting the experimental protocol. The initial induction procedure lasted for about 15 minutes and then, to ensure the quality of the maintenance of the hypnotic analgesia throughout the experiment, 2–3 minutes of hypnosis reinforcement were delivered at three other times: before starting the pain localizer protocol, before starting the empathy task protocol and in-between the two blocks of the empathy task.

### Pain localizer

During the pain localizer protocol, noxious and non-noxious thermal stimulations were delivered to the back of the right hand using a computer controlled thermal stimulator with an MRI-compatible 25 × 50 mm fluid-cooled Peltier probe (MSA Thermotest). Each thermal stimulus consisted of a ramp phase of 3 secs (increasing from a baseline value of 36 to the target temperature), followed by a plateau of 2 secs, and then return to the baseline value of the thermode^3^.

The noxious temperature was selected on an individual basis through a double random staircase procedure^12, 13^ and corresponded to stimulations strong enough to be considered painful but still bearable for a few seconds without moving^3^. Two independent staircases were presented randomly to avoid any anticipation effect between the participant’ s rating and the subsequent temperature. The initial temperatures for the two staircases were set at 40 °C and 42 °C. Within each staircase, the stimulus temperatures decreased or increased by steps of 2 °C, while smaller changes of 1 °C were used following turning points in the staircases. Using this approach, the individually defined noxious temperature varied from 45 °C to 50 °C (median 47.2 °C, standard dev. 1.91 °C) for the normal block, and from 47.5 °C to 51 °C (median 49.6 °C, standard dev. 1.32 °C) for the hypnotic analgesia block.

During the pain localizer scan, noxious and non-noxious stimulations were delivered in a pseudo-random order. There were 6 stimulations of each category (noxious and non-noxious). We used a jittered inter-stimulus interval between each stimulation ranging from 10 seconds to 14.5 seconds (average 11.7 seconds) during which the temperature was fixed at a “neutral” baseline value of 36 °C. A visual cue identical for all categories of stimuli preceded the onset of temperature increases (duration 2 seconds).

### Empathy task

Participants were presented with photographs of human hands in emotionally arousing/aversive situations that were either painless (for example, a hand holding a scalpel without being hurt) or painful (a hand being cut by a scalpel). A total of 104 distinct stimuli (52 painful and 52 painless, 768 × 768 pixels) were generated by pooling photographs from a previous study^3^ and Web search. All of these experimental stimuli depicted a right hand.

In addition, we introduced a set of 20 photographs taken from the same painful and painless categories but depicting a left hand, as well as another 20 “oddball’ pictures showing various body parts (but no hands) with no emotional component. All images were equated in luminance. To ensure equal attention to all experimental stimuli, participants were instructed to perform a go/no-go task by pressing a button with their left index only if the displayed picture depicted a left hand in the foreground. Thus, all critical experimental trials with the painful (right) hand pictures were uncontaminated by any overt motor responding.

The only instructions given to the participants related to the go/no-go paradigm, and no mention was made about perspective-taking, emotional content, or empathic processing.

Stimulus presentation during this task was divided in 4 runs (2 in the normal condition and 2 in the hypnotic analgesia condition). Each run was randomly composed of 13 painful and 13 painless stimuli as well as 5 “go” stimuli (depicting a left hand) and 5 oddball stimuli (other body parts). Each photograph was presented for 2.5 seconds with a jittered inter-trial interval ranging from 2.5 to 6 seconds. In each run, a blank trial in which an empty screen replaced the photograph was presented about every 5 stimuli, in order to optimize the baseline BOLD contrast. Within each run, stimuli were presented in randomized order.

### fMRI procedure

Participants were scanned during a single session that lasted about 60 minutes, comprising both the normal state and hypnotic condition (in random order across participants). Participants laid supine with their head maintained fixed with an ergonomic air head-pillow to minimize motion during acquisition. Visual stimuli were back projected on a screen using Matlab’s Psychtoolbox-3 for the pain localizer protocol, and Psychopy 1.80.00^36^ for the empathy (go-nogo) task. Responses were recorded using a button box (HH-2 × 4-C, Current Designs, Inc, USA).

Brain MR data were acquired on a Siemens Trio 3-T whole-body scanner for both T1-weighted anatomical images and gradient echoplanar T2*-weighted MRI images, with blood oxygenation level-dependent (BOLD) contrast. Thirty-six slices were acquired in a descending order with a 2.1 ms repetition time, an echo time of 30 ms, slice thickness of 3.2 mm, gap between slices 3.8 mm flip angle 80 °C. Acquisition of the functional data for each scanning block started after discarding the first 5 volumes.

### Behavioural data analysis

Data from individual noxious temperature, reaction times and correct responses in the go-nogo task were analyzed using the R software^37^ and plots were created by adapting code from ref. 38.

### Functional data analysis

#### Preprocessing

Functional images were preprocessed with the SPM 8 software using a standard procedure (http://www.fil.ion.ucl.ac.uk/spm/software/spm8/). For each subject and each experimental session, all volumes were first spatially realigned to the first image of the session. The T1-weighted anatomical volumes were spatially co-registered to the mean functional images resulting from spatial realignment. Functional images were then spatially normalized to the Montreal Neurological Institute (MNI) single-subject template, resampled at 2 × 2 × 2 mm voxel size, and spatially smoothed using a Gaussian kernel (8 mm full-width at half-maximum). Data for the pain localizer and the empathy task were then analyzed independently.

### Pain localizer data

#### First-level analysis

To account for the slow brain response to thermal stimuli (which peaked after 3 secs), we used a finite impulse response (FIR) analysis to determine the hemodynamic response specific to the noxious and non-noxious stimulation, without a priori on its shape or latency (similar to previous studies on thermal pain, see refs 22, 39). The six differential movement parameters were added as co-variates of no interest in order to model head movements and a high-pass filter with a cut-off period of 128 seconds was used to remove low-frequency signal drifts. The time window was set to 20 seconds allowing for an independent evaluation of the BOLD response peak in 10 successive time-bins of 2 seconds each. Individual subject F-contrasts were computed to evaluate significant effects over the entire time-window in noxious and non-noxious conditions. This procedure allowed us to identify brain areas showing the strongest reactivity to pain without any a priori concerning the onset and duration of the corresponding BOLD increase.

### Second-level analysis

For the second-level analysis of the pain localizer scan, we selected the time-bin displaying the maximal hemodynamic response across the whole participant group (i.e. 7th time-bin) and then used it to compute all contrasts between experimental conditions using a random-effect flexible factorial design. The noxious temperature values for each state and each subject was added as a covariate, allowing us to minimize activity differences reflecting temperature effects only, and conversely optimize activity differences reflecting pain perception. There were 4 experimental conditions including Noxious Normal, Non-noxious Normal, Noxious Hypno, and Non-noxious Hypno.

Statistical analyses were performed on a voxel-wise basis across the whole brain. We report activations that survive a threshold of *p* < 0.05 family-wise error (fwe) corrected for multiple comparisons across the whole brain. Small volume correction masks (sphere of 10 mm) were also used based on peak activations in the Nox > Non-Nox contrast (main effect of pain). In addition, we defined anatomical masks encompassing all regions with differential responses between noxious and non-noxious conditions that survive a threshold of *p* < 0.05 family-wise error (fwe), for later use in functional ROI analyses of the empathy task. The ROIs included the left and right insula, the ACC, SMA, thalamus and PAG.

#### Empathy task data

Trials were classified into 5 conditions: painful and painless images seen in normal state and painful and painless images seen under hypnotic analgesia with right-hand pictures, plus a general category of salient non-emotional stimuli comprising the “Go” (left hand) and oddball (non-hand pictures), in addition to trials of no interest where the participants made a mistake (i.e. pressing the button on a “No-Go” picture).

### First-level analysis

As pictures were presented with a brief duration (2.5 sec) and short inter-trial intervals (2.5–6 sec), data from this run were analyzed using the general linear model (GLM) for event-related designs as implemented in SPM. Trial time onsets from each condition were modelled using a delta function. Potential habituation effects in neural responses were accounted for by using the time modulation option implemented in SPM, creating for each active condition an additional linear regressor in which response amplitude was modulated parametrically. Each regressor was convolved with a canonical hemodynamic response function and associated with a regressor describing its first-order temporal derivatives to model variations in response onsets. The six differential movement parameters were also added as co-variates of no interest to model head movements. A high-pass filter using a cut-off period of 128 seconds was used to remove low-frequency signal drifts. There were 5 different trial types of interest, including: painful and painless images seen in normal state, painful and painless images seen during hypnotic analgesia, all with right-hand pictures; plus an additional category of salient non-emotional stimuli comprising the “Go” (left hand) and oddball (non-hand pictures). Trials of no interest where the participants made a mistake (i.e. pressing the button on a “No-Go” picture) were also modelled separately.

### Second-level analysis

A random-effect flexible factorial design was built using the contrast images obtained from the individual analyses, corresponding to each of the main comparisons of interest (effect of state and effect of picture type, with conditions and subjects as main factors.

Unless otherwise noted, we report areas showing significant effect in whole brain contrast with a statistical threshold of *p* < 0.05 fwe. The statistical significance of a priori regions of interest was further assessed using small volume correction (SVC) for multiple comparisons. The SVC masks were defined using the WFU Pickatlas toolbox (Maldjian *et al*. 2003) and clusters independently defined in the pain localizer analysis (see above) as well as main effect of painful images and main effect of noxious thermal stimuli (SVC based on sphere of 10 mm at peak activation coordinates for these 2 latter cases); with all reported activations surviving a threshold of *p* < 0.05, fwe corrected. Contrasts employed as exclusive masks in the comparison between conditions were thresholded at a liberal threshold *p* < 0.05, in order to exclude even trend level effects, with statistical significance of activation within the residual volume tested at a threshold of *p* < 0.001.

### Functional connectivity

Finally, to explore the interplay of amygdala and insula with other brain regions depending on state (Norm or Hypno), we performed functional connectivity analyses using standard a psychophysiological interaction methodology (PPI)^40^. This analysis allows for assessing the pattern of activity correlating with each seed region across all other region across the whole brain, and its changes between two conditions. Seed regions were defined using the peak coordinates of the right amygdala and left insula clusters that were identified in the group analysis for the contrast [painful images > painless images Normal state]. We then extracted these ROI time-courses from each individual when viewing negative and neutral stimuli in the normal and hypnotic conditions (taken from f-maps, averaging values over a 10 mm sphere centered on peak coordinates). Next, we remodelled the data of each participants with regressors corresponding to our 8 experimental trial categories (psychological regressors: painful images Normal state and painless images session 1 and 2 and painful images Hypnosis and painless images Hypnosis, session 1 and 2), plus the time-course from the defined volumes of interest (VOIs) over all sessions (physiological regressor), and the time-course of the VOIs during each of the 8 experimental conditions mentioned above (psychophysiological regressor). We finally entered these regressors in a second level flexible factor design.

### Subsidiary multivariate pattern analysis (MVPA)

For each of the 11 participants for which self-pain perception data were available, we used fMRI data preprocessed as in the main analysis with the exception that they were not normalized nor smoothed. MVPA was then performed on this data using the Decoding Toolbox^41^ with a cross-classification analysis design between the empathy and self-pain perception task. The classifier was first trained on the empathy task data (to define voxels activated by painful versus painless scenes) and then tested on the self-pain data (activation to noxious versus non-noxious thermal stimulation). We applied the classification kernel decoding method integrated in the Decoding Toolbox, and results were expressed in terms of accuracy minus chance scores. The resulting classification images from the single participant level were then normalized within SPM8, smoothed using a 3 × 3 × 3 kernel, and submitted to a random effect second level analysis (see Supplementary information and Supp. Fig. 1).

### Data availability

The generated statistical T maps are accessible online as 3D interactive images on NeuroVault^42^: http://neurovault.org/collections/1526/.

## Electronic supplementary material

Supplementary information
