# Author Correction: Hypnotic analgesia reduces brain responses to pain seen in others

**DOI:** 10.1038/s41598-018-35543-9

**Published:** 2018-11-16

**Authors:** Claire Braboszcz, Edith Brandao-Farinelli, Patrik Vuilleumier

**Affiliations:** 1 Laboratory for Behavioural Neurology and Imaging of Cognition, Campus Biotech, University of Geneva, 1211 Geneva, Switzerland; 2 School of Psychology, University of Plymouth, Drake Circus, Plymouth, PL4 8AA United Kingdom; 3 Department of Anesthesiology, Pharmacology and Critical Care, University Hospital of Geneva, 1211 Geneva, Switzerland; 4 Swiss Center for Affective Sciences, University of Geneva, 1211 Geneva, Switzerland

Correction to: *Scientific Reports* 10.1038/s41598-017-10310-4, published online 29 August 2017

The Acknowledgements section in this Article is incomplete.

“This project was supported by funding from the European Union’s Seventh Framework Programme under grant agreement no. 267171 as well as from the Fyssen Foundation and from the European Union’s Horizon 2020 research and innovation programme under grant agreement no. 660711. We are grateful to Corrado Corradi-Dell’Acqua, Gil Sharvit and Tomas Ros for their valuable advice on the task design and analysis.”

should read:

“This project was supported by funding from the European Union’s Seventh Framework Programme under grant agreement no. 267171 as well as from the Fyssen Foundation. This project has received funding from the European Union’s Horizon 2020 research and innovation programme under the Marie Sklodowska-Curie grant agreement No 660711. We are grateful to Corrado Corradi-Dell’Acqua, Gil Sharvit and Tomas Ros for their valuable advice on the task design and analysis.”

