# Supplementary material for: Hypnotic analgesia reduces brain responses to pain seen in others
Source: Sci Rep. 2017 Aug 29;7:9778. doi: 10.1038/s41598-017-10310-4 (PMC5575101; doi:10.1038/s41598-017-10310-4)
Supplement: Supplementary file 1 — Supplementary information [file 41598_2017_10310_MOESM1_ESM.pdf]

Hypnotic analgesia reduces brain responses to pain seen in others

Claire Braboszcz, Edith Brandao-Farinelli, and Patrik Vuilleumier

Supplementary information

Multivariate pattern analysis of nociceptive and empathic responses

While this was not the primary focus of our study, we conducted a supplementary multivariate voxel-by-voxel pattern analysis (MVPA) to further test the notion of neural overlap between brain responses to self-pain and vicarious pain. A cross-classification analysis was performed on cortical activity patterns evoked by felt and vicarious pain, in the normal condition (Corradi-Dell'Acqua et al., 2011). This analysis demonstrated a consistent overlap of activations at the voxel-by-voxel level within the left insula and the anterior cingulate cortex. This confirms that empathy for the pain of others involves brain systems that are recruited when pain is experienced by oneself and thus modulated by hypnotic analgesia.

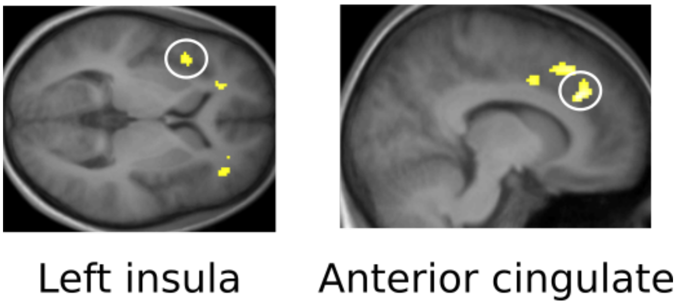

Figure 1: Results of the multi-voxel decoding cross-validation analysis. A classifier was trained on the data from the empathy task using a voxel-by-voxel pattern classification approach (MVPA) and then tested against data from the pain localizer task, in the normal condition (without hypnosis). The maps shows activations corresponding to classification accuracy minus chance score.

Region at peak	x	y	z	size(k)	P	Z values
Self-pain						
**Noxious>Non-Noxious Norm.**						
Ant. Insula L.	-36	6	0	1850	< 0.001*	6.65
Ant. Insula L.	-30	28	2	1850	< 0.001*	6.44
Post. Insula L.	-40	-2	-4	1850	< 0.001*	6.24
Post. Insula L.	-38	-20	14	192	< 0.001*	5.84
Ant. Insula R.	36	32	-2	1192	< 0.001*	6.03
Ant. Insula R.	42	12	0	1192	< 0.001*	5.89
Post. Insula R.	44	-12	14	40	0.007*	5.23
Ant. Cingulate Cortex R.	10	8	36	1131	< 0.001*	6.29
Ant. Cingulate Cortex L.	0	6	42	1131	< 0.001*	6.08
Supp. Motor Area R.	12	6	52	1131	0.001*	5.77
Thalamus R.	12	-10	12	195	0.001*	5.77
Thalamus R.	16	-18	10	195	0.007*	5.22
PAG L.	-10	-20	-12	40	0.001*	5.76
Rolandic Opercular L.	-38	-32	20	192	0.001*	5.72
Rolandic Opercular R.	50	6	12	1192	< 0.001*	5.96
Frontal Inferior Orbital R.	36	32	-2	1192	< 0.001*	6.03
SupraMarginal R.	64	-30	24	243	0.001*	5.73
SupraMarginal L.	-56	-28	22	288	0.001*	5.72
Putamen R.	24	14	6	1192	< 0.001*	6.35
Postcentral R.	60	-14	18	243	0.002*	5.46
Temporal_Inf L.	-58	-38	20	288	0.002*	5.52
Temporal_Mid L.	-58	-18	20	288	0.008*	5.21
Cerebelum R.	40	-48	-32	159	0.004*	5.38
Cerebelum R.	30	-56	-30	159	0.006*	5.25
Cerebelum R.	22	-58	-30	159	0.047*	4.79
Cerebelum R.	10	-48	-14	65	0.014*	5.08
Cerebelum L.	-4	-46	-16	65	0.008*	5.21
Cerebelum L.	-26	-54	-30	304	< 0.001*	5.83
Cerebelum L.	-44	-56	-34	304	0.002*	5.52
Vermis	0	-50	-8	65	0.013*	5.09
**Noxious>Non-Noxious Hypno.**						
Ant. Insula R.	30	18	-8	1681	< 0.001*	6.78
Ant. Insula R.	40	14	0	1681	< 0.001*	6.44
Post. Insula R.	44	-14	14	66	0.002*	5.5
Ant. Insula L.	-34	18	-6	1412	< 0.001*	6.68
Ant. Insula L.	-34	16	2	1412	< 0.001*	6.46
Ant. Insula L.	-32	26	2	1412	< 0.001*	6.18
Ant. Cingulate Cortex R.	10	6	34	1475	< 0.001*	7.2
Ant. Cingulate Cortex L.	-10	-10	44	1475	0.001*	5.77
Supp. Motor Area L.	-4	0	42	1475	< 0.001*	6.7
Thalamus L.	-16	-18	2	105	0.006*	5.25
Thalamus L.	-10	-12	-4	105	0.007*	5.24
PAG L.	-10	-20	-12	105	0.01*	5.15
Rolandic Opercular L.	-42	-34	20	363	< 0.001*	5.83
Rolandic Opercular R.	48	-30	20	487	0.001*	5.71
Putamen R.	28	4	8	1681	0.001*	5.73
SupraMarginal R.	58	-34	28	487	0.001*	5.61
SupraMarginal R.	-52	-26	20	363	< 0.001*	5.85
SupraMarginal R.	52	-22	28	487	< 0.001*	6.23
SupraMarginal L.	-48	-32	26	363	0.004*	5.35
Cerebelum R.	12	-68	-14	272	0.002*	5.48
Cerebelum R.	12	-52	-12	272	0.003*	5.45
Cerebelum R.	8	-60	-10	272	0.003*	5.41

Table 1: Self pain table of activations for contrasts of interest. Nox: noxious srimuli; Non-Nox: non noxious stimuli; (*) fwe; (**) fwe with svc. PAG:

Region at peak	x	y	z	size (k)	P	Z values
Seen pain						
**Painful images>Painless images Norm.**						
Vis. cortex L.	-14	-96	-4	540	< 0.001*	7.25
Vis. cortex L.	-16	-96	4	540	< 0.001*	6.88
Vis. cortex R.	18	-88	-6	264	< 0.001*	6.69
Thalamus L.	-4	-18	-4	186	< 0.001*	5.96
Thalamus R.	4	-18	-2	186	0.001*	5.68
Thalamus R.	8	-8	0	186	0.002*	5.48
Somatosensory R.	62	-24	44	56	0.001*	5.63
Somatosensory R.	64	-20	36	56	0.004*	5.28
Amygdala R.	28	4	-22	50	0.004*	5.3
SMA R.	12	6	64	86	0.07**	3.92
Ant. Insula L.	-30	24	8	65	0.004**	4.62
PAG L.	-8	-24	-10	28	0.013**	4.31
PAG L.	-12	-22	-10	28	0.02**	4.21
**Painful images>Painless images Hypno.**						
Brainstem	0	-38	-24	53	0.038**	4.12
Vis. cortex L.	-14	-98	-8	100	< 0.001*	6.20
Vis. cortex R.	18	-92	-8	135	< 0.001*	6.05
**Excl. masking [painful images>painless images Norm.] by [Painful images>Painless images Hypno.]**						
Ant. Insula L.	-28	22	8	50	0.006**	4.6
Thalamus L.	-4	-20	-4	667	< 0.001**	5.8
Thalamus R.	4	-18	-2	667	< 0.001**	5.68
Thalamus R.	8	-8	0	667	< 0.001**	5.48
Thalamus R.	8	-8	0	667	< 0.001**	5.48
PAG L	-10	-24	-12	667	0.01**	4.48
Amygdala R.	30	2	-18	139	< 0.001**	5.16
Amygdala R.	30	2	-24	139	0.004**	4.68
Amygdala R.	26	2	-22	139	0.004**	4.68
Brainstem L.	-8	-18	-6	667	0.035**	4.14
Brainstem R.	14	-18	-6	667	0.006**	4.58

Table 2: Activation table for seen pain in hypnosis, (*) fwe; (**) fwe with svc; Vis.: visual; PAG: periaqueductal grey; SMA: supplementary motor area; Ant. anterior; Post: posterior

Region at peak	x	y	z	size(k)	P	Z values
Oddball and "go" stimuli						
**[Go+Oddball stimuli Normal] > [Painful images + Painless images Normal]**						
Sensorimotor R.	44	-18	56	207	< 0.001*	6.13
Sensorimotor R.	48	-18	46	207	0.001*	5.72
Sensorimotor R.	40	-26	58	207	0.01*	5.13
**[Go+Oddball stimuli Hypno] > [Painful images + Painless images Hypno]**						
Sensorimotor R.	46	-22	54	1036	< 0.001*	6.32
Sensorimotor R.	32	-24	54	1036	< 0.001*	6.32
Cingular med. R.	6	-12	48	351	< 0.001*	7.22
SMA R.	12	-6	50	351	< 0.001*	6.91
SMA R.	6	2	40	351	0.001*	5.67
Post. Insula R.	50	-14	14	151	< 0.001*	6.08
Rolandic Opercular R.	50	-20	20	151	0.002*	5.45
Post. Insula R.	40	-2	12	151	0.005*	5.27
Parietal Inf. L.	-52	-56	40	72	0.001*	5.58
Parietal Inf. L.	-48	-54	50	72	0.005*	5.28
Angular gyrus R.	48	-70	36	73	0.002*	5.48
Inferior parietal lobule R.	44	-60	38	73	0.012*	5.1
Middle temporal gyrus R.	62	-18	-18	30	0.002*	5.44
Middle temporal gyrus R.	62	-26	-14	30	0.01*	5.14
**Interaction [Painful images Norm > Painful images Hypno] > [Go+Oddball stimuli Norm > Go+Oddball stimuli Hypno]**						
Ant. Insula L.	-34	12	2	206	0.497**	3.35
Ant. Insula L.	-32	12	-8	206	0.542**	3.31
SMA R.	6	12	50	103	0.042**	4.18
SMA R.	4	10	54	103	0.045**	4.16
SMA R.	2	14	52	103	0.068**	4.05
Ant. Insula R.	32	24	8	107	0.104**	3.92
Ant. Insula R.	36	26	6	107	0.138**	3.84
Ant. Insula R.	34	24	-2	107	0.223**	3.68
Amygdala R.	34	6	-20	36	0.001	3.14
Amygdala R.	36	2	-24	36	0.002	2.95
Amygdala R.	38	-4	-26	36	0.002	2.88

Table 3: Activation table in response to "go" and oddball stimuli, (*) fwe; (**) fwe with svc.
